# MAPPING THE LITERATURE FOR THE PHYSICAL ACTIVITY SCALE FOR THE ELDERLY (PASE): A SCOPING REVIEW

**DOI:** 10.1093/geroni/igae098.3486

**Published:** 2024-12-31

**Authors:** Cassandra D’Amore, Lexie Lajambe, Noah Bush, Sydney Hiltz, Justin Laforest, Isabella Viel, Qiukui Hao, Marla Beauchamp

**Affiliations:** McMaster Univeristy, Hamilton, Ontario, Canada; McMaster Univeristy, Hamilton, Ontario, Canada; Fairway Physiotherapy, Thunder Bay, Ontario, Canada; McMaster University, Hamilton, Ontario, Canada; Choice Health Centre, Halifax, Nova Scotia, Canada; McMaster University, Hamilton, Ontario, Canada; McMaster University, Hamilton, Ontario, Canada; McMaster University, Hamilton, Ontario, Canada

## Abstract

The Physical Activity Scale for the Elderly (PASE) is a commonly used and recommended self-report measure of physical activity in older adults. This scoping review aimed to map the nature and extent to which the PASE has been used in community-dwelling older adults, including the evidence for its psychometric properties. Review eligibility criteria included: i)community-dwelling older adults (60+ years), ii)physical activity integrated into study aims and measured with the PASE, and iii)published in English. Pairs of independent reviewers screened abstracts, full-texts, and extracted data. Where possible, weighted mean PASE scores were calculated for subgroups based on age, sex, and clinical population. Seven electronic databases were searched in January 2023; 4,124 studies were screened, and 232 articles from 35 countries were included. Most studies were completed in high-income countries (86.4%) and North America (49.57%). A variety of clinical conditions were included (n=21), the most common populations being osteoarthritis (n=13), Parkinson’s disease (n=11), and cognitive impairment (n=7). Psychometric properties for ten versions of the PASE were reported. All versions demonstrated acceptable test-retest reliability. Evidence for construct validity reported moderate correlations with self-reported physical activity and fair to moderate correlations with accelerometry-derived activity. Pooled means were reported in graphs and forest plots by age, sex, and clinical populations. The PASE was widely used across geographical locations and culturally adapted to several populations. Some psychometric properties have been evaluated; however, further research is required to examine responsiveness and predictive validity. The weighted mean PASE scores presented can improve interpretability of scores in similar populations.

